# Positive cultures and clinical outcomes in septic patients: be aware of the influence from patient selection and the in-hospital confounders

**DOI:** 10.1186/s13054-019-2602-7

**Published:** 2019-10-29

**Authors:** Romain Jouffroy, Benoît Vivien

**Affiliations:** SAMU de Paris, Service d’Anesthésie Réanimation, Hôpital Universitaire Necker - Enfants Malades, Assistance Publique - Hôpitaux de Paris, and Université Paris Descartes - Paris 5, Paris, France

To the Editor:

We read with great interest the paper published by Nannan Panday et al. [1], who reported that, first, culture-positive sepsis patients have a higher mortality rate than those with culture-negative and, second, culture-positive sepsis patients more often have multiple organ systems affected during the sepsis episode. Firstly, we compliment the authors for this very interesting study.

Nevertheless, to our opinion, some methodological issues deserve their attention. Firstly, the database used for the analysis comes from another study (PHANTASi trial) [2], in which, eligible patients were those with a suspected of (severe) sepsis and septic shock [2]. Thus, we cannot be certain that all included patients have sepsis rather than an alternative diagnosis. The authors do not report how many patients were septic or not; we cannot rule out that patient selection could be the biggest issue directly affecting the study results [1]. Indeed, the blood culture results may be different if included patients were septic or not. This may also partly contribute to the negativity of the PHANTASi trial [2].

Secondly, the variables included in the multivariate analysis (age, group allocation, hospital location, source of infection, antibiotics at home and total amount of blood cultures drawn) do not consider the potential cofounders of the in-hospital phase. Mortality is strongly affected by the in-intensive care unit and in-hospital length of stay due to their potential complications [3]. For example, during the hospital stage, patients, especially elderly patients, may be affected by a limitation of care and/or nosocomial infection. Beyond these 2 methodological issues, we should keep in mind that, apart from source control, sepsis mortality is not only affected by antibiotherapy, but also by a bundle of care among which hemodynamic optimization plays an important role [4, 5].

Nevertheless, we fully agree that, contrary to septic shock, early identification of sepsis, especially those at risk of unfavourable evolution, is particularly difficult in the prehospital setting where the diagnosis is based on a list of non-specific physiological variables. The additional use of biomarkers, blood lactate measurement to assess severity and procalcitonin to confirm the bacterial origin of sepsis may be useful in order to define which patients should benefit from early prehospital antibiotic administration. Such a strategy enhancing better patients’ selection could help the physician in the decision-making process and predict the real impact of early antibiotherapy.

## Data Availability

Not applicable.
