# Supplementary material for: Positive cultures and clinical outcomes in septic patients: be aware of the influence from patient selection and the in-hospital confounders
Source: Crit Care. 2019 Oct 29;23:332. doi: 10.1186/s13054-019-2602-7 (PMC6819441; doi:10.1186/s13054-019-2602-7)
Supplement: Related texts

# Reply: Authors’ response

**Authors:** Rishi S. Nannan Panday, Eline M. J. Lammers, Prabath W. B. Nanayakkara

Dear editor,

We’ve read with interest the letter to the editor by Jouffroy et al. We would like to thank the authors for their valuable input and hereby our reaction to their comments.

Firstly, the authors state that perhaps not all patients included in our study had sepsis, but they may have an alternative diagnosis. As described in our study, all patients met SEPSIS-2 criteria at inclusion and retrospective chart analysis of all charts by a panel of experts (consisting of two acute physicians and one infectious disease specialist) was performed in order to exclude patients with an alternative diagnosis. This resulted in 1% of patients in the intervention group and 2% in the control group with a different diagnosis. Although these patients were included in both the PHANTASi trial and our (sub) study, we believe that such small numbers will not have had an effect on the outcomes [1, 2].

Secondly, Jouffroy et al. raise the point that potential in-hospital confounders, such as length of stay and nosocomial infections, should be part of the multivariate regression model used in our study. However, we believe hospital length of stay may be part of the causal pathway, i.e. patients with positive cultures have a higher sepsis severity, are more critically ill and therefore have to stay in hospital longer. As described by Jager et al., including covariates that are to be part of the causal pathway of the outcome in a model may lead to over-adjustment [6]. Indeed, nosocomial infections are common in sepsis patients, although studies report a low attributable risk of mortality caused by nosocomial infections [7]. We agree that superinfections should be considered as a covariate in future studies.

Apart from source control and antibiotic therapy, outcomes in septic patients are certainly effected by supportive care; however, hemodynamic optimization would mainly play a large role in septic shock patients, which were only approximately 4% of patients in our study cohort.

We agree with Jouffroy et al. that use of additional biomarkers, such as pro-calcitonin and lactate, in the (pre) hospital setting would guide emergency medical service personnel and clinicians to make a bacterial origin of infection more or less plausible. Currently, the level of the pro-calcitonin is not routinely measured in sepsis patients in the Netherlands, but this should be considered in the future. However, the role of procalcitonin in the emergency department setting is still a matter of debate.
